# Supplementary material for: Timely excision of prophage Φ13 is essential for the Staphylococcus aureus infectious process
Source: Infect Immun. 2025 Sep 3;93(10):e00314-25. doi: 10.1128/iai.00314-25 (PMC12519783; doi:10.1128/iai.00314-25)
Supplement: Supplemental material — Fig. S1 to S4; Tables S1 and S2. [file iai.00314-25-s0001.pdf]

Timely excision of prophage Φ13 is essential for the *Staphylococcus aureus* infectious process

Poupel *et al*. IAI00124-25

Supplementary Information

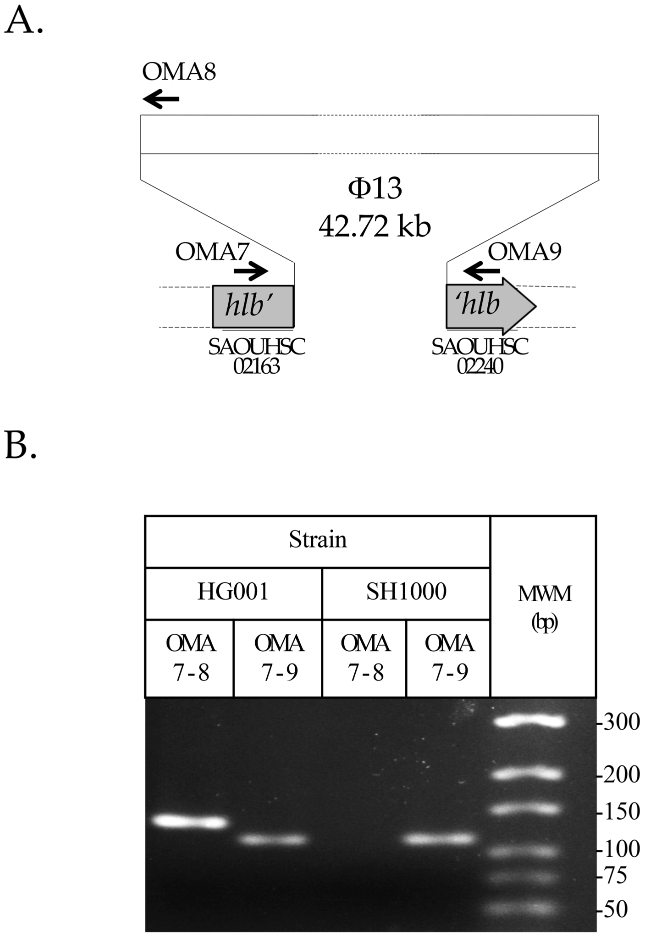

Supplementary Data Fig. 1
PCR-based detection of phage Φ13 insertion and excision

A: The Φ13 chromosomal insertion locus in strain HG001 (not to scale). The Φ13 *att*P sequence is homologous to the *att*B site located within the *hlb* gene encoding β-hemolysin. Φ13 insertion thus results in disruption and inactivation of the *hlb* gene. Specific primers used to quantify the proportion of lysogenic bacteria are indicated, pair OMA7-OMA8 is specific for Φ13 insertion and pair OMA7-OMA9 for the absence of Φ13.
B: Specific amplicons obtained by PCR with the OMA7-8 and OMA7-9 primer pairs using genomic DNA from either the SH1000 strain, cured for Φ13 (114 bp), or from the Φ13 lysogen strain HG001 (132 and 114 bp).

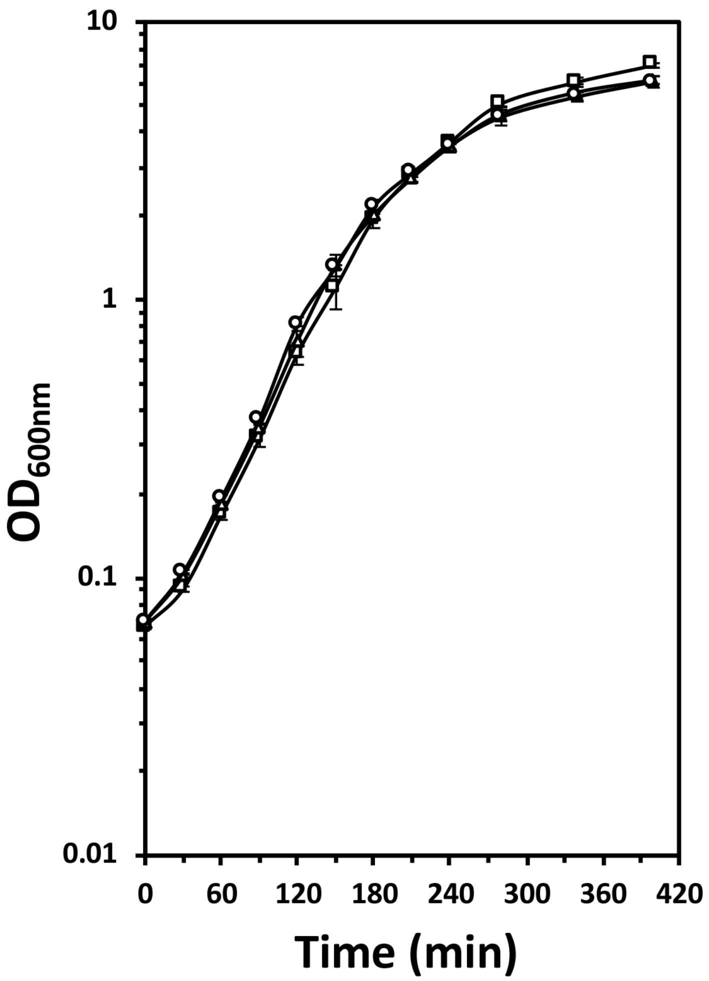

Supplementary Data Fig. 2 Growth curves of *S. aureus* strains HG001 and the ΔΦ13 and Δ*int* mutants.

Bacterial cultures were grown overnight, inoculated in TSB at a calculated OD_{600nm} of 0.05 and incubated at 37°C with aeration. Optical densities were followed over a 6.5 hour period. Results are shown as the mean and standard deviation of three independent growth curves.

Doubling times (http://www.doubling-time.com/compute.php) were calculated during the exponential growth phase (between 60 min and 150 min) and gave values of approximately 32 min for each strain. Strains: HG001 (□); ΔΦ13 (△); Δ*int* (○).

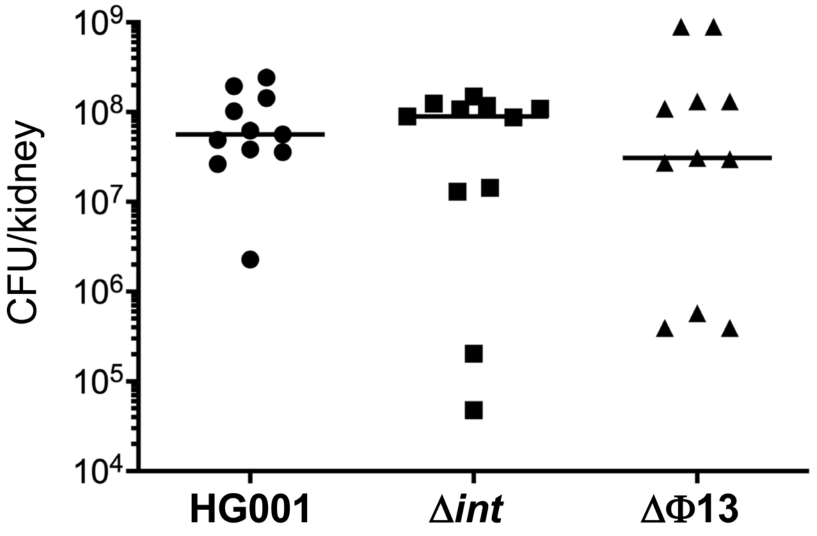

Supplementary Data Fig. 3 Kidney colonization following infection by *S. aureus*
RjOrl:SWISS mice were infected with the *S. aureus* HG001 wild type strain or the Δ*int* and ΔΦ13 derivatives by intravenous injections with a sub-lethal bacterial load (2.10^7 cfu/injection). Six days post-infection, mice were sacrificed, kidneys were harvested and CFU counts carried out. A total of 11 mice were studied in two independent experiments and the data are compilation of the bacterial counts.

A.

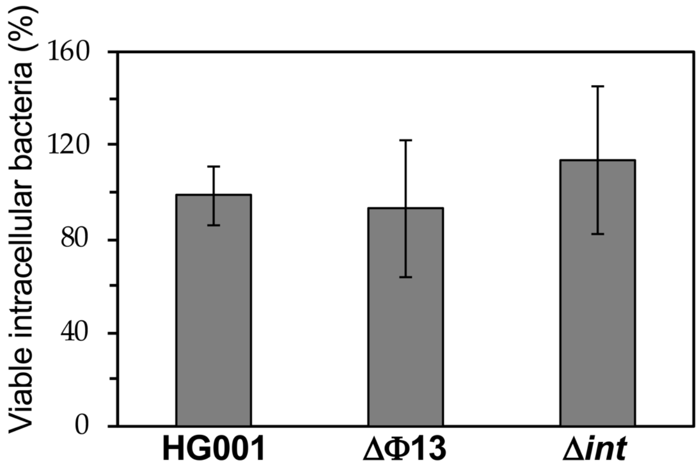

B.

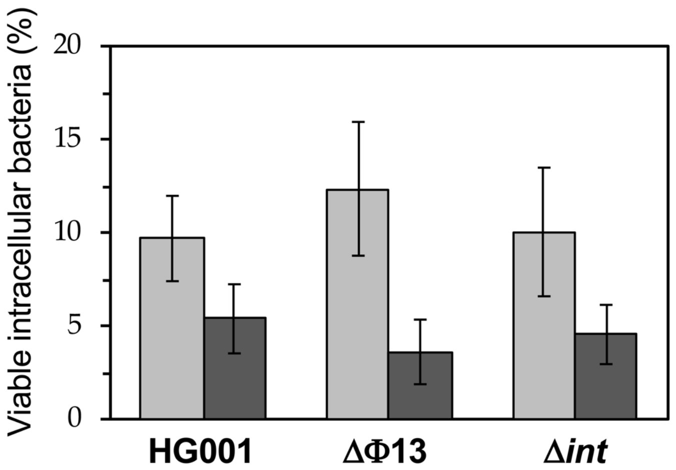

Supplementary Data Fig. 4 Impact of Φ13 on internalization and survival within THP-1 neutrophils.

PMA-activated THP-1 cells were infected with either the HG001 wild type strain or the ΔΦ13 and Δ*int* mutants with a moi=10.

A: Viable intracellular bacteria were quantified two hours post-infection to determine the efficiency of internalization.

B: Viable bacteria were quantified 24 H (light grey) and 48 H (dark grey) post-infection to determine the capacity of each strain to survive within neutrophils.

Results are presented as means and SEM of 3 biological replicates.

Supplementary Data Table 1: Strains and plasmids used in this study

Strain or plasmid	Description	Source or reference
***E. coli* strains**		
DH5α	F^- Φ80*lac*ZΔM15 Δ(*lac*ZYA-*arg*F) U169 *rec*A1 *end*A1 *hsd*R17 (r_K–, m_K+) *pho*A *sup*E44 λ– *thi*-1 *gyr*A96 *rel*A1	Invitrogen Life Technology
***S. aureus* strains**		
RN4220	Restriction-deficient transformation recipient strain	[1]
SH1000	NCTC 8325-4 *rsbU*+	[2]
HG001	NCTC 8325 *rsbU*+	[3]
ST1327	HG001Δ*int*	pMAD-*int* → HG001
ST1330	HG001ΔΦ13	pMAD–Φ13 → HG001
ST1346	HG001Δ*int* pMK4P*prot*	pMK4P*prot* → ST1327
ST1347	HG001Δ*int* pMK4P*prot-int*	pMK4P*prot-int* → ST1327
ST1348	HG001Δ*int* Δ*hlb*	pMAD-*hlb* → ST1327
ST1387	HG001Φ13-*aad*(9)	pMAD Φ13-*aad*(9) →HG001
ST1435	HG001Δ*int* *hlb*comp	pMAD-*hlb*comp → ST1327
**Plasmids**		
pMAD	Allelic exchange vector	[4]
pMAD–Φ13	pMAD derivative for Φ13 deletion	This study
pMAD-*int*	pMAD derivative for *int* deletion	This study
pMAD-*hlb*	pMAD derivative for *hlb* deletion	This study
pMAD-*hlb*comp	pMAD derivative for *hlb* complementation	This study
pMK4P*prot*	Vector for P*prot*-dependent gene expression (complementation)	[5]
pMK4P*prot-int*	Plasmid used for *int* deletion complementation	This study

1 Kreiswirth, B. N. *et al.* The toxic shock syndrome exotoxin structural gene is not detectably transmitted by a prophage. *Nature* **305**, 709-712, (1983).

2 Horsburgh, M. J. *et al.* σ^B modulates virulence determinant expression and stress resistance: characterization of a functional *rsbU* strain derived from *Staphylococcus aureus* 8325-4. *J. Bacteriol.* **184**, 5457-5467, (2002).

3 Herbert, S. *et al.* Repair of global regulators in *Staphylococcus aureus* 8325 and comparative analysis with other clinical isolates. *Infect. Immun.* **78**, 2877-2889, (2010).

4 Arnaud, M., Chastanet, A. & Debarbouille, M. New vector for efficient allelic replacement in naturally nontransformable, low-GC-content, gram-positive bacteria. *Appl. Environ. Microbiol.* **70**, 6887-6891, (2004).

5 Archambaud, C., Gouin, E., Pizarro-Cerda, J., Cossart, P. & Dussurget, O. Translation elongation factor EF-Tu is a target for Stp, a serine-threonine phosphatase involved in virulence of *Listeria monocytogenes*. *Mol. Microbiol.* **56**, 383-396, (2005).

Supplementary Data Table 2 : Oligonucleotides used in this study

Name	Sequence	Description
Construction of pMAD derivatives		
OSA418	TGGGATCCTAGTCTTAAATCTATATCACTAATACC	Φ13 upstream and
OSA419	CAGAATTCAACAATATAACTCGCTTCGTCC	downstream fragment
		(*Bam*HI/*Eco*RI)
OSA411	AAAGGATCCTACAAGTGTAGACGTTGAATGGTGGTGAG	*int* upstream fragment
OSA412	CGTGAATTCATGCGTTCCCTCCTCAAAATTGGC	(*Bam*HI/*Eco*RI)
OSA413	AGTGAATTCTTTAGGGACCCATTAGGGACTCCAAACCC	*int* downstream fragment
OSA414	ATATACATCCCATGGCTTAGGTTTTTCAGTCAC	(*Eco*RI/*Nco*I)
OSA442	ATCGGATCCATTGTGTATGGAACTTGGTAATTTGC	*hlb* upstream fragment
OSA443	TTTGTCGACCATTATCACTCCTTTTATATAGCTTAC	(*Bam*HI/*sal*I)
OSA444	TGTGTCGACTGCTCAACTAACTAATAACTTGCTTCG	*hlb* downstream fragment
OSA445	AATGAATTCAACAATACAAATGTGACTGACTTTGCACC	(*Sal*I/*Eco*RI)
OSA508	TTTGGATCCTTACTGACTGCACTTAAATATTATTTAGAC	*aad*(9) insertion upstream
OSA509	GACGTCGACGGAGGATTTTAAATGTTTAAGG	fragment (*Bam*HI/*sal*I)
OSA510	AAACCATGGCTTTCCGTCACTCTTTAATTGG	*aad*(9) insertion
OSA511	AAGAGATCTTGAAACTTTTTCTCTCATTTAGTGC	downstream fragment
		(*Nco*I/*Bgl*II)
OSA299	ATGTCGACGGATCTTCACCTAGATCCTTTTGAC	*aad*(9) amplicon
OSA470	TTCCCATGGTACAAATTGTTTCACTAAATTAAAGT	(*Sal*I/*Nco*I)
OSA418	TGGGATCCTAGTCTTAAATCTATATCACTAATACC	*hlb* and its upstream region
OSA573	TCAGTCGACAACAATATAACTCGCTTCGTCC	(*Bam*HI/*Sal*I)
OSA574	CCAGTCGACAGACTTTTAACATAAAATTACTTATC	*att*L downstream region of □13
OSA575	AAAAGATCTAAGTTTAACCAATATGTTGCTCG	(*Sal*I/*Bgl*II)
pMK4Pprot complementation		
OSA446	AACGGATCCCTAAATTTTGGGTAGCCCGCCTACCC	*int* gene amplification
OSA447	AACGTCGACACAACAGTATTTATTGGGTTTGGAGTCC	(*Bam*HI/*Sal*I)
qRT-PCR experiments		
OMA7	GAAAGATGATACTGATTTGA	Amplicon specific for Φ13
OMA8	AACCTTGATATTATGCTGTT	presence (OMA7-OMA8)
OMA9	TAAGAAGATTGTCCGATTA	and absence (OMA7-
		OMA9)
OSA438	TACAAACAACTTCAAACTGTTGTGC	
OSA439	AAACATGCGTGGAAAACACAGAGG	

OMA25	CAAATGGGAGCCTTTTCTTA	Amplicon specific for Φ12 presence (OSA438-OSA439) and absence (OSA438-OMA25)
OMA18<br>OMA19<br>OMA20	AAGGAAGTTTAAGACGATGAA<br>AAACGCATTAAATCAACG<br>TGGCTACAACGCATATTAC	Amplicon specific for Φ11 presence (OMA18-OMA19) and absence (OMA18-OMA20)
